# Monitoring, characterization and control of chronic, symptomatic malaria infections in rural Zambia through monthly household visits by paid community health workers

**DOI:** 10.1186/1475-2875-13-128

**Published:** 2014-03-31

**Authors:** Busiku Hamainza, Hawela Moonga, Chadwick H Sikaala, Mulakwa Kamuliwo, Adam Bennett, Thomas P Eisele, John Miller, Aklilu Seyoum, Gerry F Killeen

**Affiliations:** 1 Ministry of Health, National Malaria Control Centre, Chainama Hospital College Grounds, off Great East road, P.O. Box 32509, Lusaka, Zambia; 2 Liverpool School of Tropical Medicine, Vector Biology Department, Pembroke Place, Liverpool L3 5QA, UK; 3 Centre for Applied Malaria Research and Evaluation, Tulane University School of Public Health and Tropical Medicine, New Orleans, LA, USA; 4 Partnership for Appropriate Technology (PATH) in Health Malaria Control and Evaluation Partnership in Africa (MACEPA), Chainama Hospital, Lusaka, Zambia; 5 Ifakara Health Institute, Biomedical & Environmental Thematic Group, P.O. Box 53, Ifakara, Morogoro, United Republic of Tanzania

**Keywords:** Malaria, Community health worker, Surveillance, Passive and active case detection, Treatment with ACT, Rapid diagnostic tests

## Abstract

**Background:**

Active, population-wide mass screening and treatment (MSAT) for chronic *Plasmodium falciparum* carriage to eliminate infectious reservoirs of malaria transmission have proven difficult to apply on large national scales through trained clinicians from central health authorities.

**Methodology:**

Fourteen population clusters of approximately 1,000 residents centred around health facilities (HF) in two rural Zambian districts were each provided with three modestly remunerated community health workers (CHWs) conducting active monthly household visits to screen and treat all consenting residents for malaria infection with rapid diagnostic tests (RDT). Both CHWs and HFs also conducted passive case detection among residents who self-reported for screening and treatment.

**Results:**

Diagnostic positivity was higher among symptomatic patients self-reporting to CHWs (42.5%) and HFs (24%) than actively screened residents (20.3%), but spatial and temporal variations of diagnostic positivity were highly consistent across all three systems. However, most malaria infections (55.6%) were identified through active home visits by CHWs rather than self-reporting to CHWs or HFs. Most (62%) malaria infections detected actively by CHWs reported one or more symptoms of illness. Most reports of fever and vomiting, plus more than a quarter of history of fever, headache and diarrhoea, were attributable to malaria infection. The minority of residents who participated >12 times had lower rates of malaria infection and associated symptoms in later contacts but most residents were tested <4 times and high malaria diagnostic positivity (32%) in active surveys, as well as incidence (1.7 detected infections per person per year) persisted in the population. Per capita cost for active service delivery by CHWs was US$5.14 but this would rise to US$10.68 with full community compliance with monthly testing at current levels of transmission, and US$6.25 if pre-elimination transmission levels and negligible treatment costs were achieved.

**Conclusion:**

Monthly active home visits by CHWs equipped with RDTs were insufficient to eliminate the human infection reservoir in this typical African setting, despite reasonably high LLIN/IRS coverage. However, dramatic impact upon infection and morbidity burden might be attainable and cost-effective if community participation in regular testing could be improved and the substantial, but not necessarily prohibitive, costs are affordable to national programmes.

## Background

A relatively large proportion of malaria infections are only mildly symptomatic, especially in endemic countries where acquired immunity moderates both parasitaemia and pathology [1-3]. Even in areas with only modest, seasonally sporadic transmission where little immunity exists among the human populations, the many mildly symptomatic infections that persist chronically, often at sub-patent parasite densities below thresholds of detectability, are responsible for sustained malaria transmission [4,5]. As early as the 1930s, these populations have been targeted for treatment with anti-malarial drugs as a control measure for malaria transmission, particularly during the era of the Global Malaria Eradication Programme initiated in the 1950s [6-8]. Due to the emergence of drug resistance, which was attributed to mass drug administration campaigns [9-11] and evidence that the impact on transmission may be limited, the use of therapeutic approaches to control transmission rather than burden was not considered as effective [6,8,12,13] until recently, when interest in malaria elimination was rejuvenated [13-15].

Currently curative drugs are predominantly targeted at symptomatic individuals based on passive detection of acute infections among patients seeking care for fevers through the formal and informal health system [16-18]. Strategies for control of human-to-mosquito transmission by providing chemotherapy to chronic parasite carriers are now being revisited as they may be complimentary to front-line interventions for preventing mosquito-to-human transmission, such as long-lasting insecticidal nets (LLINs) and indoor residual spraying (IRS) of houses, to further accelerate declines in malaria burden [19-21] in areas of low and seasonal transmission [8,13,22].

However, most of the infected fraction of the human population only exhibits sub-acute symptoms so they do not always obtain medical care, even where it is readily available [23,24]. These carriers of chronic, mildly symptomatic malaria infection thus act as the silent reservoir of infection because even low, often sub-patent levels of parasitaemia are sufficient to infect mosquitoes [25-27]. Additionally, asymptomatic individuals risk developing chronic anaemia and symptomatic malaria [28,29]. Across all levels of transmission, the majority of infections and onward transmission to mosquitoes occurs in older age groups, even though the young often harbour the highest parasite densities, because the former comprise the bulk of the human population and vector biting rates increase with host biomass and therefore age [20,30,31]. In such populations, many individuals remain parasitaemic and infectious for one or more years [32-34]. Thus, in order to eliminate human-to-mosquito transmission with therapeutic drugs, all cryptic or asymptomatic infections within a human population must be successfully terminated, necessitating comprehensive coverage of targeted communities [20,21,28,35].

Essentially two broad strategies for taking malaria chemotherapy beyond routine case management have been described: mass drug administration (MDA) or mass screening & treatment (MSAT), historically referred to as mass blood examination [20,36]. MDA entails administering anti-malarials to every traceable consenting member of a population, regardless of whether their malaria infection status is known or whether they exhibit symptoms [13,20,36], while MSAT targets only confirmed parasitaemic individuals after parasitological testing for infection status [20,36]. MDA necessitates comprehensive coverage and failure may accelerate the spread of drug-resistant strains selected by strong but incomplete selection pressure on the parasite populations [37,38]. MSAT has been proposed as a better alternative because treatment is limited to those diagnostically confirmed to be infected, thus lowering treatment costs and risks of selection for resistance [39,40]. However, the major limitation of MSAT lies in the challenge of detecting low-density infections, which contribute substantially to the reservoir of infection [40]. With increasing availability of more sensitive, scalable malaria rapid diagnostic tests (RDTs) that can be used by non-specialist community based health workers in low-resource settings, MSAT is now logistically feasible [38,39,41,42].

The concept of community-based management of malaria stems from the recognition that human resource deficits amongst clinically-trained professional cadres are commonplace, so extending service delivery beyond centralized health facilities, by mobilizing through community health workers (CHW) will be required to improve access to appropriate management of uncomplicated malaria [43,44]. CHWs have demonstrated the capacity to effectively diagnose malaria with RDTs and provide treatment according to the locally relevant policy and guidelines [41,42,45,46]. The community-based diagnosis and treatment approach has also been shown to be cost-effective [47,48], improve delivery of malaria case management overall [49-51], is well accepted by communities [52-54] and also provides a potentially valuable population–wide platform for monitoring trends in human parasitaemia [55].

However, this approach remains grossly underutilized and understudied, with only 15 million RDTs utilized at community level globally, mostly in India [56]. This study therefore evaluated the effectiveness of paid CHWs providing improved access to blood screening and treatment services to community members, not only when they self-reported because they felt ill, but also through regular monthly active visits to their homes.

## Methods

### Study areas

**Figure 1:**
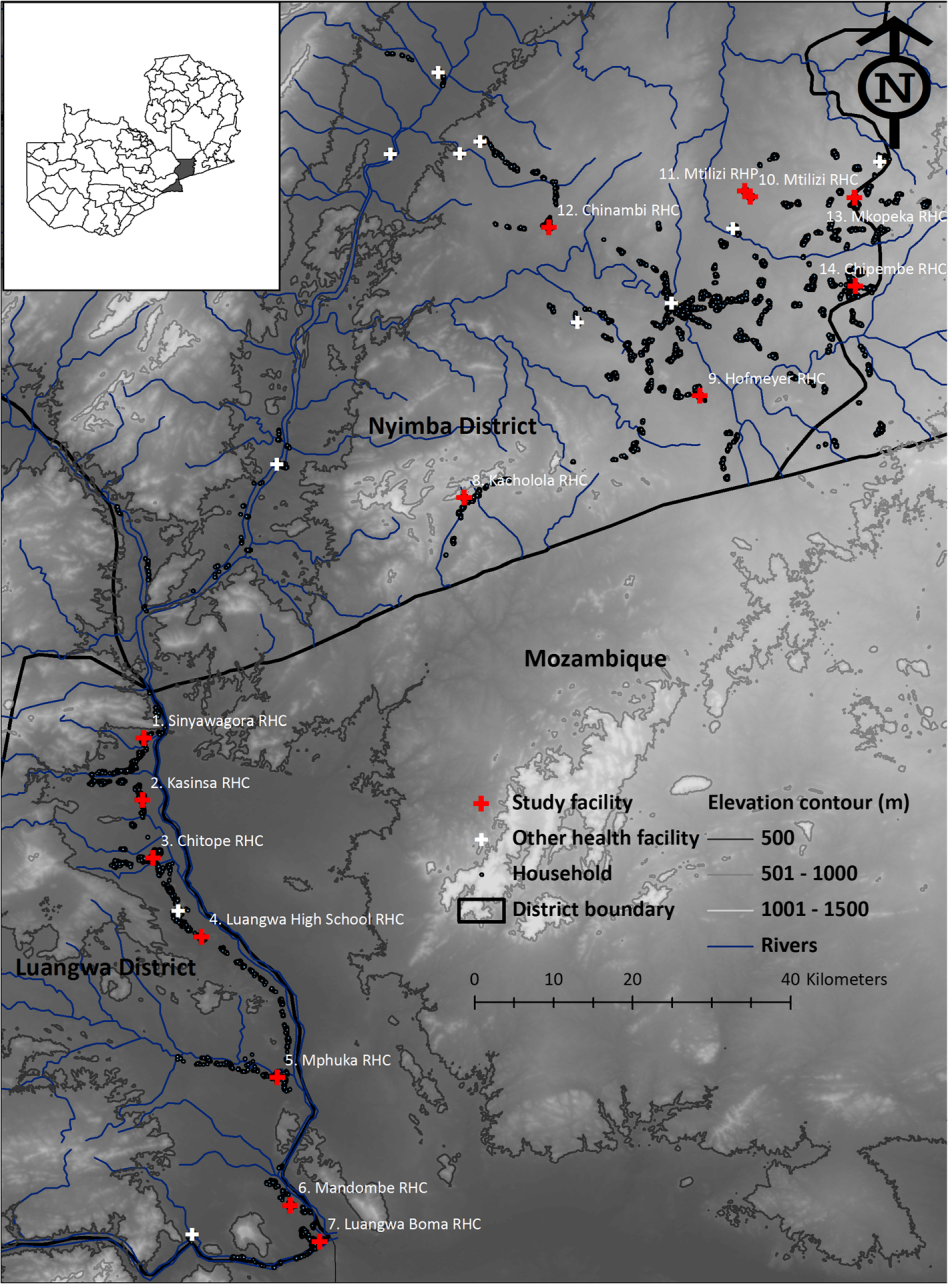
Map indicating location of health facilities and associated catchment populations enrolled in the study.

The study was conducted in two adjacent rural districts of Zambia, Luangwa District in Lusaka province and Nyimba District in Eastern province (Figure 1) where perennial transmission of *Plasmodium falciparum* is predominantly mediated by *Anopheles funestus*[57,58] at an entomologic inoculation rate (EIR) of approximately 70 infectious bites per unprotected person per year (Sikaala CH, Chinula D, Chanda J, Hamainza B,Mwenda M, Mukali I, Kamuliwo M, Lobo N, Seyoum A and Killeen GF, personal communication).

Nyimba is located 350 kilometres east of the capital city Lusaka and covers an area of 10,943 km^2^ with a population of approximately 86,000 residents, most of whom are involved in agriculture as their main livelihood. Nyimba is drained by three perennial rivers the Luangwa and its two tributaries the Lunsemfwa and the Lukusashi, as well several streams. Nyimba spans a range of altitudes from 400 m to 1,200 m along the Luangwa valley with temperatures averaging 30°C and mean annual rainfall of 1,000 mm [59]. The population of Nyimba is served by 17 health centres and one first-level hospital located in the centre of Nyimba town.

Luangwa district is located at the confluence of the Zambezi and Luangwa rivers, 325 kilometres south-east of Lusaka. The population of the Luangwa is 25,000 residents, most of whom are settled along the river where the main economic activities are fishing and agriculture. The district covers a surface area of 3,468 km^2^ and lies 350 to 500 metres above sea level. Luangwa has an average temperature in above 35°C and a mean annual rainfall of approximately 800 mm [59]. Luangwa has nine health centres, of which two have inpatient services, one of which is located in the district central business area at Luangwa Boma and another at Katondwe Mission located about 40 km further north.

### Community health workers

In Zambia, community health workers (CHWs) are a formally accepted part of the health service delivery system and are selected by the communities they serve. In order to qualify, they must reside within the community, be literate in English, and be available and willing to provide basic care to their communities. Once selected, they are trained for a period of six weeks in basic primary healthcare, mainly focusing on aspects of prevention and treatment of common ailments as part of their health promotion duties. The major disease focus areas are the simple forms of malaria, pneumonia and diarrhoea. When care is sought from the CHWs, the patient’s symptoms are assessed and the CHWs follow the guidelines for diagnosis and treatment. The CHWs are also trained how to recognize clinical danger signs and facilitate referral of these cases to the HFs for further management [60]. Each CHW is attached to the HF nearest to their community and is normally responsible for approximately 500 of the resident inhabitants. The clinically-trained HF staff are responsible for supervising and mentoring the CHWs, and for supplying all their equipment and consumables. The CHWs that participated in this study were selected from available CHWs in their specific catchment areas and recruited through their supervising HFs. These recruited CHWs were remunerated at a rate of 350 Zambian Kwacha (approximately US$71.58) per month. A total of three CHWs per cluster (42 active CHWs) were selected to participate in the study and re-trained for a week in malaria diagnosis (use and interpretation of RDTs), treatment (prescription and dosage of AL), referral procedures (recognition of danger signs) and reporting.

### Study design

This longitudinal study was conducted, from January to December 2011. In each study district, seven clusters of approximately 165 consenting households (average number of resident members per household was estimated at approximately six [61], each of which was selected and enrolled in order of proximity to the public-sector HF that defined the centre of the cluster. All members of the household were eligible to participate except pregnant women and children below six months of age. The exclusion of these groups was stipulated in accordance with national guidelines that CHWs are not allowed to manage any condition in these select groups. Each cluster received the standard Ministry of Health interventions for malaria control in Zambia, which included long-lasting insecticidal nets (LLINs), diagnosis by either microscopy or rapid diagnostic tests (RDTs), treatment with artemether-lumefantrine (AL) and intermittent presumptive treatment with sulphadoxine-pyrimethamine. The LLINs were distributed through mass distribution campaigns in 2006, 2008 and again 2009 [62], following a project-based distribution to selected villages in Luangwa in 2005 [63] and routine delivery for pregnant women and under five children through the antenatal clinics at each of the health facilities in the study [64,65]. Intermittent presumptive therapy in pregnancy IPTp) was also routinely provided through the antenatal clinics with a target of 3 doses during each pregnancy [64,65].

### Data collection

Each CHW recorded all patient diagnoses and responses to pre-set questions in a malaria register book which detailed RDT outcomes, as well as details of age, symptoms, use of IPTp or LLINs, and treatment of their house with IRS in the previous six months. Information on access and use of interventions was collected at an individual level among all participants for those who gave consent to participate in the study. Active case detection was achieved through monthly home visits by the CHWs to each enrolled household within their assigned catchment areas, during which all consenting and assenting members were screened for malaria infection using HRP2-based RDTs from ICT Diagnostics (ICT Malaria Pf cassette test). Between these visits, passive case detection was accomplished by encouraging those who had symptoms to seek care from either their assigned local CHW or the nearest public sector HF. Individuals that escorted patients who sought care were also tested if they were identified as members of the study clusters. During both active and passive visits, all study participants found to be positive for malaria by RDT received standard AL treatment for uncomplicated malaria. Those found to be febrile and negative by RDT through either active or passive testing systems were referred to the nearest healthcare facility. In both the active and passive systems, the CHWs reported weekly summaries via mobile phone short messaging system of the number of patients tested, RDT test results, AL and RDT stock status and the numbers of treatments of each pack size (6, 12, 18 or 24 tablets) they had dispensed.

### Data management and statistical analysis

The principal data sources for this study were the CHW malaria registers and health facility reports sent to NMCC through the malaria rapid reporting system platform [66] via the Airtel™ mobile phone network in the two districts. To ensure data quality and full disaggregation, the line-listed data from the malaria registers were double-entered and verified, reconciled and then cleaned following descriptive frequency analysis of the distributions of values for each variable. Statistical analysis was accomplished using SPSS version 20 (IBM) and R version 2.14.1 augmented with the lattice, Matrix and LME4 packages.

### Association of malaria infection with age, sex, symptoms, interventions, cluster and season

Generalized linear mixed models (GLMM) were used to evaluate the association between observed RDT-determined malaria infection status as a binary dependent variable with an intercept, age category (<1, 1 to 4, 5 to 10, 11 to 14, 15 to 24, 25 to 44 and >45 years of age), sex, access to an LLIN, use of an LLIN, having slept in a house that had been treated with IRS in the previous six months, and the three seasons (hot and wet from December to April, cool and dry from May to August, and hot and dry from September to November) as categorical independent variables. All models included household identity number nested within CHW catchment nested within cluster, as well as date of participant contact, as random effects, with the exception one model lacking an intercept in which cluster was treated as a categorical independent variable to determine the mean diagnostic positivity of each cluster. The final model presented in Additional file 1 was selected by building the model through adding explanatory variables to the model one at a time, assessing improvements of the goodness of fit using log-likelihood tests and retaining only those parameters that either had significance in the model or which significantly improved the goodness of fit as per principle of parsimony [67].

### Association of malaria infection with clinical symptoms

While several symptoms were indeed positively associated with *P. falciparum* infection, these were excluded from the final model that captures the effects of most of the variables presented in Additional file 1 simply because they are an effect, rather than a cause, of malaria so they cannot be regarded as underlying independent determinants of malaria risk. Instead, the association of these symptoms with malaria infection was assessed as follows, using separate GLMMs treating the presence or absence of each symptom as the binary dependent variable and malaria infection diagnostic result as a binary, categorical, independent explanatory variable (Table 1). Each model also included age, sex and season as additional categorical independent variables and individual study participants nested within CHW catchment, nested again within clusters as random effects. Each model was selected to include only variables, which were significant or significantly improved the goodness of fit, as described above for the models of malaria infection risk (Additional file 1).

**Table 1:** **Association of symptoms of illness with RDT positivity, age and seasonality**^**iii iv**^

**Symptom**	**Passive surveillance**		**Active surveillance**	
	**OR [95% CI]**^**c**^	**P**^**d**^	**OR [95% CI]**^**c**^	**P**^**d**^
Fever	5.98[5.79,6.16]	<0.001	14.63[14.55,14.71]	<0.001
History of fever	2.16[1.93,2.40]	<0.001	2.86[2.77,2.95]	<0.001
Headache	2.31[2.15,2.47]	<0.001	6.83[6.77,6.90]	<0.001
Cough	0.74[0.58,0.90]	<0.001	1.85[1.77,1.93]	<0.001
Diarrhoea	1.47[1.15,1.79]	0.017	2.04[1.86,2.21]	<0.001
Vomiting	3.01[2.74,3.27]	<0.001	6.61[6.43,6.80]	<0.001
Chest pain	0.88[0.56,1.20]	0.447	1.47[1.31,1.64]	<0.001
Breathing problems	1.78[−8.61,12.16]	0.914	7.99[6.47,9.52]	0.008
Other symptoms	0.87[0.46,1.28]	0.510	0.98[0.81,1.15]	0.809

^iii^c – odds ratio with 95% confidence intervals, d – p-value.

^iv^The association of clinical symptoms with malaria infection as determined by RDT positivity was determined by logistic regression with GLMMs controlling for age and seasonality as fixed effects and for date, individual nested in CHW and CHW nested in cluster location as random effects.

### Cost per case diagnosed and treated

The approximate cost of diagnosis and treatment of malaria parasite infection through either the CHW or health facility systems was calculated separately. For the CHWs, this was split into two arms: active and passive. The costs incorporated into our calculations were personnel time, RDTs, microscopy where available, anti-malarial drugs and sundry maintenance, transport and consumables. Personnel time included an assumed 30% full time equivalent (FTE) contribution to malaria diagnosis and treatment by all personnel based at the HF and the CHWs were assumed to have allocated 90% and 10% FTE contributions to the active and passive service delivery arms. The cost of diagnosis was estimated by the multiplying the unit cost (US$0.31 per RDT and US$1.30 per microscopy test) [68,69] of each diagnostic method by the number of tests done. The cost of drugs was estimated by multiplying the total number of treatments dispensed by the average Zambian malaria programme unit cost of US$1.38. Annual remuneration costs for all the CHWs are calculated based on their monthly remuneration of US$71.58 (ZMW 350) per month per CHW and the number of person months for which they were engaged over the course of the study. Annual costs of remuneration for health facility staff were collated from the medium term expenditure frameworks of the two district medical offices, totalling US$281,150 (ZMW 1,377,600). Total personnel and other facility maintenance costs were calculated by dividing the total number of times all study participants/patients were appropriately diagnosed and treated. Subsequent estimates of cost per case diagnosed and treated were made by adding personnel time, costs of diagnosis and treatment per case and facility maintenance costs. It was not possible to estimate capital costs for either the CHW or health facility systems, so these were excluded from these calculations. Calculations of directly observed costs were based on those recorded over the six months from April to September 2011 when all the CHW and HF were fully functional but before IRS was introduced as a possible confounding effect. A projected per capita cost estimate for a year was then estimated by doubling these six month cost estimates. Furthermore a projected potential cost per capita for implementing such an equivalent CHW programme but complete compliance of the catchment population with monthly testing was also calculated. Annual per capita cost estimates for the actual and enhanced-participation scenarios were also calculated for scenarios in which transmission is reduced to levels where treatment costs were negligible.

### Population attributable fraction of symptoms to malaria infection

The proportion of cases of each specific clinical symptom identified within the population by the CHWs that could be attributed to RDT-detectable malaria infections was estimated as follows [3]:

$$\lambda =p\left(R-1\right)/R$$

Where *λ* is the attributable fraction, *p* is the diagnostic positivity and *R* is the odds ratio of symptom associated with malaria infection. This formula was separately applied to both the active and passive surveillance data obtained through the CHWs.

### Ethical approval

Ethical approval was obtained from the University of Zambia, Biomedical Research Ethics Committee (Reference 004-05-09) and the Research Ethics Committee of the Liverpool School of Tropical Medicine (Approval 09.60). Authority to conduct the study was also obtained from the Ministry of Health in Lusaka, Zambia.

## Results

### Rates of participation and testing through health facilities and community health workers

**Figure 2:**
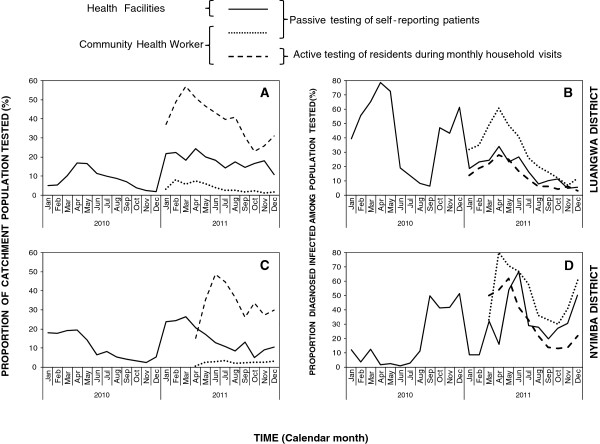
Proportion of catchment population tested (A and C) and diagnostic positivity for malaria infection among residents (B and D) in Luangwa (A and B) and Nyimba (C and D).

From a combined catchment population of 77,754 for Luangwa and Nyimba, there were approximately 75,791 outpatients visits attended to at the 14 enrolled HFs in 2011. Seasonal patterns of testing for malaria at HFs were roughly similar and peaked at approximately the same time of the year in both districts, with an overall mean rate of testing for malaria infection of 56%. Mean monthly testing rates were 36.9% (range = 0% to 100%) for patients in Luangwa and 69.3% (range =0% to 100%) in Nyimba (Figure 2A and C) so the proportion of patients in which malaria was suspected and tested for was approximately twice as high in Nyimba as Luangwa.

A total of 14 population clusters centred around these HFs, with enrolled populations that ranged from 753 to 1,243, were established and followed up over a period of one full calendar year in 2011 from January to December for Luangwa District, and April to December for Nyimba district. A total of eight and 12 monthly rounds of active household visit surveys were conducted by each CHW in Nyimba and Luangwa, respectively, to test and treat all consenting residents within these population clusters. A total population of 17,543 individuals participated by consenting to testing during the active monthly household visits, of whom 20% were under the age of five. During the same period a total of 3,804 individuals, of whom 24% were under the age of five, sought care and were tested when they self-reported to the CHW, the results of which were then recorded as a passive surveillance indicator (Additional file 1). Slightly more females participated in both the active and passive visits than males (Additional file 1), presumably because females were more accessible during the active household visits, and sought care more frequently from CHWs through their passive service provision role, than males because they spent most of their time at home [70]. The CHWs referred a total of 577 and 631 patients detected through passive and active surveillance systems, respectively, to the health facilities for further management. The main reasons provided for referral were the inability by the CHWs to manage some of the conditions presented to them by the patients, RDT positive results for pregnant women, lack of patient improvement while on malaria treatment and insufficient AL stocks in the hands of that CHW for him or her to provide treatment directly. Over the same period 42,389 and 932 suspected malaria cases were tested by RDT and microscopy, respectively, and 20,794 were treated for malaria through the health facilities in these clusters.

Introduction of CHWs for screening and treating of residents captured a higher proportion of the populations they covered than the HFs they were based near to, overwhelmingly through active monthly visits to the household rather than passive reporting (Figure 2A and C). This can be readily explained by the fact that these 14 HFs covered a total catchment population of 77,754 people, equivalent to 58.8% of the combined population of the two districts, while a total of 42 CHWs were assigned a total of 17,543 people or only 13.3% of the combined population of the two districts. The introduction of this CB extension of primary healthcare services had no obvious impact upon attendance rates at health facilities in Luangwa, and a simultaneous drop in HF attendance in Nyimba resembled seasonality patterns from the previous year (Figure 2A and C). Thus no obvious impact of CHW services upon the rates of reporting of suspected malaria to the local health facility was apparent.

**Figure 3:**
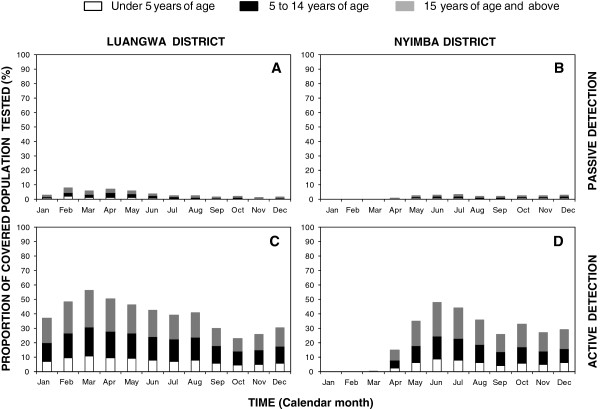
Age and proportion of covered population tested for malaria infection each month as contacted passively (A and B) and actively (C and D) by community health workers in Luangwa (A and C) and Nyimba (B and D).

The proportion of enrolled participants who actually consented to testing during active household visits rose rapidly and then peaked at approximately half during March in Luangwa (Figure 2A) and June in Nyimba (Figure 2C). The proportion of enrolled individuals who sought care and were passively tested for infection by CHWs was generally far lower, only exceeding 5% in an early peak in Luangwa but not in Nyimba (Figure 2A and C). In both CHW survey arms, older children (5–14 years) and the adults (≥15 years) comprised the highest proportion of those tested while young children (<5 years) comprised a minority, presumably because they comprise a small demographic proportion of the overall population (Figure 3).

**Figure 4:**
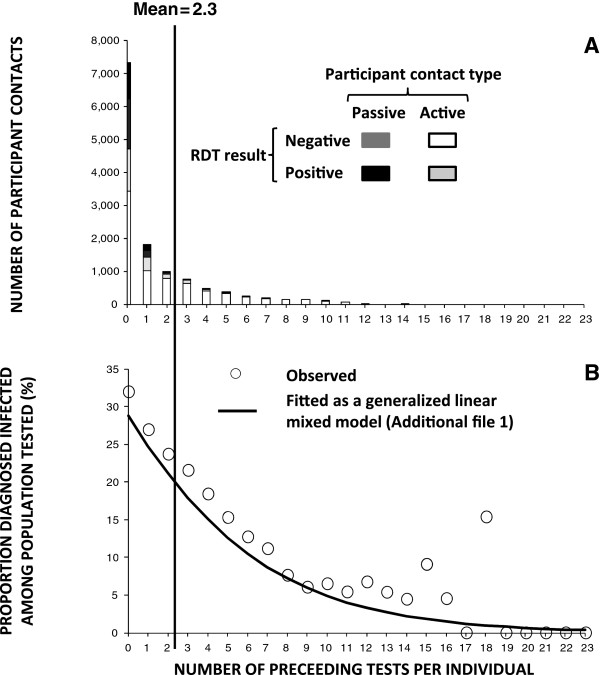
Frequency histogram of the number of study participant contacts for each total number of preceding malaria infection tests by community health workers per individual study participant (A) and the relationship between the proportion of those participants diagnosed as being infected and the cumulative number of diagnostic tests for malaria infection per individual participant (B).

Overall, less than 20% of the enrolled population was tested more than once by CHWs through either the active or passive surveys but the mean number of tests per individual (2.3) was much higher than the median (1 [range = 1 to 24]) because the frequency distributions for the numbers of times individuals were tested through either mechanism were highly skewed (Figure 4A). The average number of tests per individual participant through both surveillance systems combined was understandably somewhat higher in Luangwa (4.4) than in Nyimba (3.2) because they were operational for 12 months in the former but only eight months in the latter. The number of passive patient contacts per individual through self-reporting to CHWs ranged from 0 to 24 times in both districts over the course of the study period, with a median of only once, and an average of 1.2 times per individual. The number of active patient contacts per individual through monthly household visits by CHWs ranged from 1 to 12 times in both districts over the course of the study period, with a median of only once, and an average of 2.6 times per individual.

### Comparative rates of malaria infection diagnosis by health facilities and community health workers

Overall, the majority of occasions when residents sought care through the passive detection systems of the HFs (44%) and CHWs (57.5%) did not have malaria parasite infections (Figure 2B and D). Nevertheless from the total study population, more than 14,000 uncomplicated malaria infections were identified by CHWs over the course of 2011 with the vast majority (84.1%) of these being detected through the active household visits rather than through self-reporting to a CHW for passively offered service (Additional file 1). While the HFs detected even more malaria infections over the course of the same year (37,204), these were drawn from much larger catchment populations so the overall rate of detection of cases of malaria infection per head of population covered was highest for the active surveys of the CHWs, followed by passive surveys at the HFs and CHWs (1.16,0.40 and 0.14 diagnostically confirmed malaria infections per person per year) (Table 3). The overall incidence rates for detected and diagnostically confirmed malaria infections, broken down by district were 1.13 and 1.51 cases per person per year for Luangwa and Nyimba, respectively.

**Figure 5:**
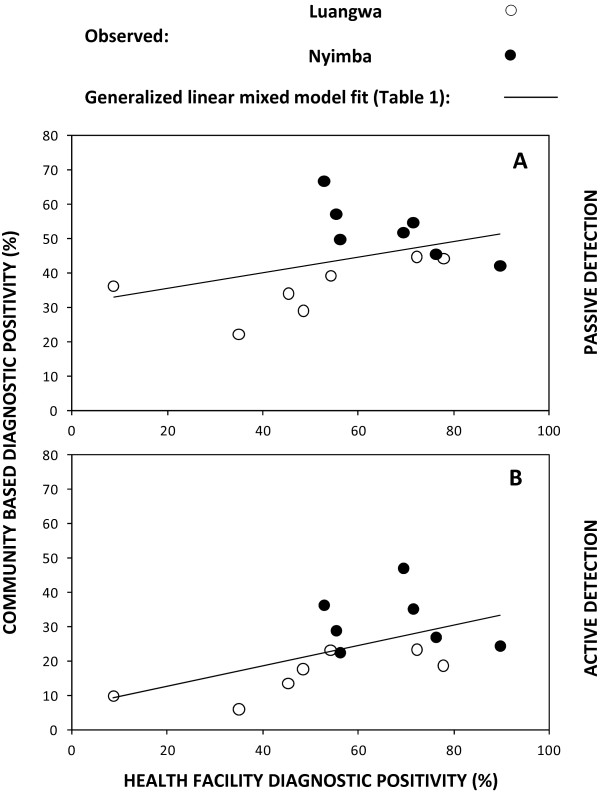
Association of diagnostic positivity for malaria infection among patients attending health facilities with diagnostic positivity recorded by community health workers through passive (A) and active (B) participant contacts.

The overall diagnostic positivity, or proportion of diagnostic tests which confirmed malaria infection, was generally far higher among patients seeking diagnosis and treatment through the routine services offered passively by either the HF or the CHWs than among those screened actively through the monthly household visits of the CHWs (Additional file 1, Figure 2B and D). This was presumably because self-reporting patients obviously present a sample that is strongly biased towards those who are actually ill at the time. Diagnostic positivity observed at the healthcare facilities fluctuated seasonally, peaking at the end of the rainy season in April and May and reaching its lowest point at the end of the dry season in September and October, with a mean of 17.2% in Luangwa (range 4.8% to 34.1%) and 31.0% in Nyimba (range 8% to 67%). The wide range of diagnostic positivity in these study sites is comparable to what has been observed in other malarious parts of Zambia [71,72] and may be considered reasonably representative of the range of transmission across most endemic parts of the country. Considerable geographical heterogeneity was also observed in the diagnostic positivity rates obtained through the CHWs, especially those from their active household surveys that were less biased towards infected individuals, with the lowest being in the two most urbanized clusters of Luangwa District (Additional file 1). Seasonal patterns of diagnostic positivity at HFs, expressed as the proportion of all patients tested diagnostically with RDTs or microscopy who were confirmed to be infected, differed appreciably between the two districts with no particularly consistent similarities or dissimilarities from 2010 to 2011 (Figure 2B and C). The seasonality patterns of diagnostic positivity among residents tested by CHWs closely paralleled those tested by HFs in Luangwa and even preceded them by a month or two in Nyimba (Figure 2) where access to HFs was more challenging, especially during the rains. Furthermore, the estimated mean diagnostic positivity of both passive and active surveillance of the CHWs were strongly and positively correlated with those observed through passive surveillance at the HFs across both districts (Figure 5) and were, therefore, highly consistent with each other as measures of malaria infection. Interestingly, diagnostic positivity rates reported by CHWs were much more closely associated with those reported by HFs in Luangwa than in Nyimba (Figure 5) where access to HFs is far more difficult for this more scattered population.

### Demographic, geographic and vector control determinants of malaria infection burden

Malaria infection among residents tested by the CHWs was associated with age, sex, season, geographical location and coverage with vector control in the form of LLINs and IRS, as well as the number of times each individual had been tested previously and, in most cases, treated for malaria (Additional file 1). Malaria infection burden among patients self-reporting to the CHW through the passive surveys peaked in exactly the same age category as those tested during their active monthly household visits (Additional file 1), confirming that essentially the same population was being monitored by both systems. Risk of infection peaked in older children and was least among infants and the oldest adults and females were slightly at less risk than males (Additional file 1). Malaria infection probability was far higher in the hot and wet season, and the cool and dry season, than in the hot and dry season (Additional file 1).

The majority of study participants who reported using an LLIN when they were tested by a CHW (72% (2,738/3,804) for passive contacts and 70.1% (12,295/17,543) for active contacts) had lower diagnostic positivity, consistent with the protective effect expected. However, individuals living in the 9 clusters that were treated with IRS towards the end of 2011, whose houses were actually sprayed, had higher diagnostic positivity, even when time, location and household effects were controlled for (Additional file 1). Rather than conclude that IRS actually increases malaria risk, it may be presumed that these estimates from best-fit models are probably a spurious artefact arising from endogeneity caused by logically and deliberately biased deployment of IRS to areas within each cluster with highest disease burden by the District Medical Offices tasked with implementing malaria control activities. Specifically, the IRS teams in both districts deliberately started spraying the most isolated villages at the fringes of the enrolled population clusters first so that these could be completed before arrival of the rains and associated limited access.

### Association of malaria infection with clinical symptoms of illness

**Figure 6:**
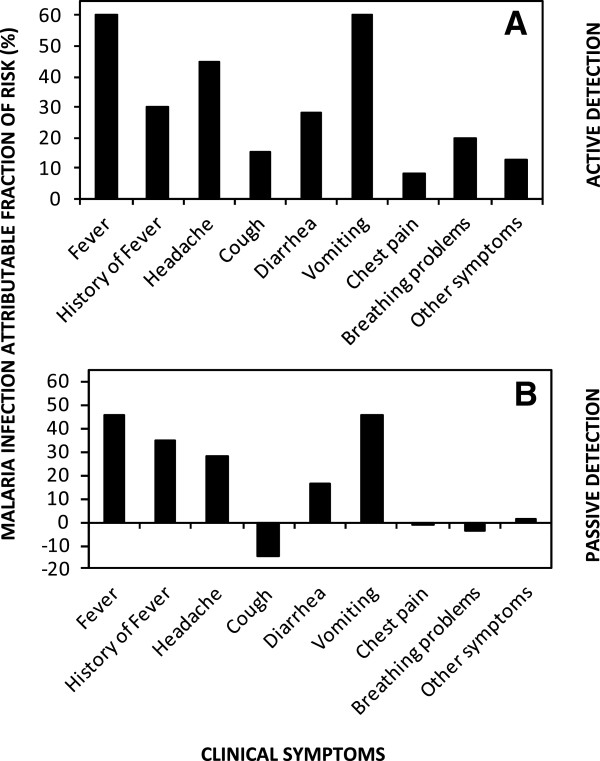
Fractions of risk for reported clinical symptoms which are attributable to malaria infection detected by community health workers through active (A) and passive (B) contact events.

A substantial proportion of all residents who reported no symptoms whatsoever were found to carry malaria parasite infection; 12% (5,123/42,881) and 27% (286/1,062) of the active and passive contacts, respectively. Discussions with CHWs confirmed that essentially all asymptomatics who were tested through passive contacts were those friends, relatives and caregivers who had escorted a patient to see the CHW and were also tested during such a visit. The overall number and proportion of all patient contacts which were classified as asymptomatic malaria infection detected by CHWs was approximately twice as high among residents tested through in active surveillance [8.8% (5,123/58,500)] rather than passive [5.4% (286/5,261)]. The proportion of confirmed malaria cases identified through active monthly surveys by CHWs who apparently exhibited no symptoms whatsoever was only 43% (5,123/11,851), confirming that most detectable malaria infections are chronic, but nevertheless associated with substantial, if non-severe, symptoms at the time they are surveyed. Malaria infection was associated with all specifically assessed symptoms, and even with the “other symptoms” category among residents screened during active household visits by CHWs, and most of these associations could also be detected using data collected passively from self-reporting patients (Table 1). The symptoms most strongly associated with malaria infection were fever, a history of fever in the last month, headache and vomiting, with the former being the highest reported in both surveillance arms (Table 1). The reverse was also found to be true as all symptoms were associated with RDT-detected malaria infection in both active and passive CHW surveys, except for breathing problems and sundry other symptoms, using the passive surveys data with limited sample size (Table 1). More than half of all cases of fever and vomiting, and more than a quarter of all cases with history of fever, headache and diarrhoea, among residents tested during active CHW visits to their households were attributable to malaria infection (Figure 6). The positive association of cough with detectable infection in the active visits, contrasting with a negative association in passive surveys that is more difficult to rationalize (Table 1, Figure 6), may reflect an interactive effect upon patient reporting rather than the manifestation of the symptom itself, resulting in under-reporting of cough among patients reporting to CHWs because they were infected with malaria.

### Association of malaria infection status and clinical symptoms of illness with malaria testing frequency

**Figure 7:**
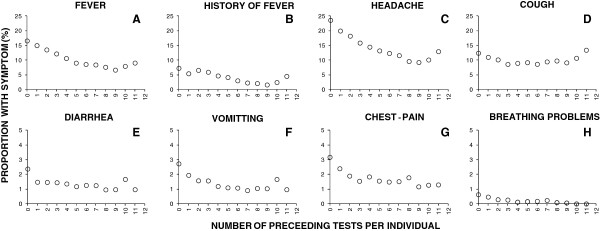
Relationship between the proportion of participant contacts with community health workers in which they experienced fever (A), history of fever (B), headache (C), cough (D), diarrhea (E), vomiting (F), chest pain (G) and breathing problems (H) and the cumulative number of preceding diagnostic tests for malaria infection per individual participant.

Only one study participant was tested 24 times by CHWs during both active and passive visits combined. Diagnostic positivity for malaria was negatively associated with the number of times that participant had been previously tested, and in most cases, treated (Additional file 1, Figure 4B). For the small proportion of individuals who received 12 tests during a single calendar year, diagnostic positivity through active surveys was estimated to be 5.4% in their last test, compared with 32% for those tested for the first time. Diagnostic positivity was therefore proportionally 83% lower for the 12th test of those who participated at least that often, and this trend towards lower diagnostic positivity continued downward for who were tested even more frequently (Figure 4B). A similar phenomenon was observed with regard to manifestation of symptoms, with much lower rates of occurrence observed for all reported clinical symptoms except cough among individuals who had been repeatedly tested and treated for malaria (Figure 7). Interestingly, even when RDT-diagnosed infection status is accounted for by adding this independent variable to the models depicted in Figure 7, the number of times an individual had been previously tested remained predictive of fever (P <0.001), history of fever (P <0.001), headache (P < 0.001), diarrhoea (P < 0.001), chest pain (P < 0.001), breathing problems, vomiting (P < 0.001) and other symptoms (P < 0.001). Taken at face value, these observations appear to suggest that screening and treatment may not only reduce probability of infection with malaria at detectable parasite densities (Figure 4B), but also persistent sub-patent infections that contribute to symptoms of illness despite parasite densities too low to be detected (Figure 7). However, testing frequency was not assigned to distinct treatment groups or experimentally controlled in any other way so these associations are purely observational and causality cannot be directly inferred. For example, these observations might also be explained by co-association of testing frequency, malaria infection and symptoms of illness with unrecorded health-conscious behaviours that are not accounted for in the model described in Additional file 1. Indeed, the test results at first active visit did seem to slightly influence of the number of subsequent tests so the trends observed in Figures 4B and 7 should be cautiously interpreted: individuals whose first test yielded a negative result had a slightly greater mean number of tests over the course of the study (4.65 ± 0.03 versus 4.11 ± 0.05, P < 0.001 by GLMM).

Despite the impressive negative association of repeated testing and treatment with the probabilities of infection and symptoms among individuals, no dramatic impact upon these parasitological and clinical outcomes were obvious and a total incidence rate of 1.7 detected infections per head of population persisted (Figure 2, Table 3). As illustrated in Figure 4A, the mean number of times participants were tested was 2.3, so even if the relationship between number of preceding tests and diagnostic positivity is causal, rather than merely co-associated, too few participants were tested regularly enough for any dramatic impacts to be observed at population level: Those who had the mean number of tests would be expected to maintain a mean diagnostic positivity of 23.7% at the end of the year, only 17.9% lower proportionally than those tested only once (Figure 4B). Even if direct impact of testing and treatment upon infection probability is assumed, and comprehensive monthly testing could be achieved in the future, this would still be expected to leave sufficient levels of parasitaemia at population level to maintain endemic transmission (Figure 4A and B) [73]. The intensity of persisting transmission reflected in the measured EIR, despite considerable levels of vector control, is also reflected in measured rates of re-infection among humans: Over the course of the study period, CHWs detected as many as eight malaria infections in a single study participant detected through passive surveillance, while the maximum was nine infections in a single participant as detected through active surveillance (Figure 4A).

### Adherence of CHWs to diagnosis and treatment guidelines

**Figure 8:**
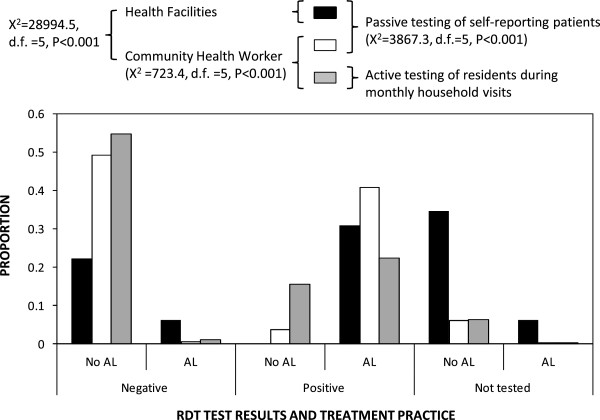
Community health worker and health facility staff treatment and diagnostic practice in relation to national guidelines.

Patterns of diagnosis and treatment differed between patients seeking care at HFs or from CHWs, as well as residents consenting to being tested by CHWs during their active household visits (Figure 8, Table 2). Adherence to national guidelines for diagnosis and treatment were generally good among CHWs with 78% of all contacts that resulted in an RDT test being followed by an appropriate decision to treat or not (Figure 8). The remainder was primarily accounted for by diagnostically confirmed cases of malaria infection that could not be treated because the CHW had run out of the drug, and also because small proportions of patients were treated in the absence of a diagnostic result or despite a negative diagnostic result (Figure 8, Table 2). More worryingly, only 53% of patients attending HFs were tested and then treated or not treated appropriately to the test result, primarily because a substantial proportion were neither tested nor treated but also because small proportions were treated in the absence of a test or despite a negative test (Figure 8, Table 2). So, consistent with reports from other settings in Zambia [41,42,46,74] and beyond [49,75,76], the CHWs had greater adherence to policy guidelines on treatment practices in relation to diagnostic test results than specialist staff at HFs (Figure 8, Table 2). The proportion of patient contacts resulting in a negative RDT test result, or assessed only clinically without a confirmatory diagnostic test, that were treated with AL were both at least six times higher at HFs than CHWs (Figure 8). CHW provision of treatment to patients with a negative test result was twice as high in the active compared with passive but in both cases this occurred only very rarely. All confirmed cases of malaria infection reporting to health facilities received treatment with AL and the same was true for 92% of those identified passively by CHWs but only 59% among those identified through active household visits (Table 2, Figure 8). In most cases where CHWs did not provide treatment for RDT-positive patients, this was because they lacked AL and those patients were referred to the HFs to collect curative drugs.

**Table 2:** **Compliance to diagnostic and treatment policy – Community based and facility based (April to September 2011)**^**v**^

		**Facility based**			**Community based**									**Community & facility based**		
		**Passive**			**Passive**			**Active**			**Passive and active**			**Passive and active**		
**Al dispensed**		**No**	**Yes**	**Total**	**No**	**Yes**	**Total**	**No**	**Yes**	**Total**	**No**	**Yes**	**Total**	**No**	**Yes**	**Total**
**Tested by**	**Test result**															
**Rdt**	**Negative**	11,024	3,046	14,070	1,518	16	1,534	24,878	517	25,395	26,396	533	26,929	374,20	3,579	40,999
	**Positive**	0	15,306	15,306	111	1,260	1,371	7,012	10,149	17,161	7,123	11,409	18,532	7,123	26,715	33,838
**Microscopy**	**Pegative**	204	55	259	n/a	n/a	n/a	n/a	n/a	n/a	n/a	n/a	n/a	204	55	259
	**Positive**	0	262	262	n/a	n/a	n/a	n/a	n/a	n/a	n/a	n/a	n/a	0	262	262
**Rdt or microscopy**	**Negative**	11,228	3,101	14,329	1,518	16	1,534	24,878	517	25,395	26,396	533	26,929	37,624	3,634	41,258
	**Positive**	0	15,568	15,568	111	1,260	1,371	7,012	10,149	17,161	7,123	11,409	18,532	7,123	26,977	34,100
**Not tested**		17,541	3,158	20,699	185	3	188	2,845	18	2,863	3,030	21	3,051	20,571	3,179	23,750
**Total**		39,997	40,496	80,493	3,443	2,555	5,998	66,625	21,350	87,975	70,068	23,905	93,973	110,065	64,401	174,466

^v^Treatment practice based on confirmatory RDT result was assessed between April and September as this was the period of time when all the CHW based active & passive surveillance and the health facility based passive system were all fully functional.

### Testing and treatment service delivery, cost and cost-effectiveness

As detailed in Table 3, expenses associated with personnel and commodities are important components of the overall cost of providing malaria diagnosis and treatment services through either HFs (49%) or CHWs (25%). Sundry transport and maintenance expenses also contributed substantially to the overall cost of the HFs but not the CHWs. The overall cost of HFs was three-fold higher than the CHW approach but was less expensive than CHWs per head of population covered, because the HFs were assigned to cover far larger catchment populations while the CHWs simply provided far more frequent testing and treatment service to each resident through the active surveys conducted through monthly household visits (Table 3). If the same frequency of testing were implemented in a pre-elimination scenario [77,78] where improved vector control reduced drug treatment requirements to negligible levels, this would save only a quarter of the overall costs of providing these HF or CHW services. Because of the lower personnel, transport and maintenance costs of the CHWs, combined with their better compliance with national guidelines, both the passive and active services provided by the CHWs were almost twice as cost-effective in terms of cost per diagnostically confirmed case identified and treated.

**Table 3:** **Observed and potential cost-effectiveness of cases appropriately diagnosed and treated**^**vi vii vii ix**^

	**Health facility**	**Community based**		
**Directly observed process indicators over 6 months**		**Passive**	**Active**	**Total**
Covered community based cluster or facility catchment	77754	17543	17543	17543
Diagnostic tests carried out over six months	29897	2652	42556	45208
Diagnostically confirmed and treated cases of malaria infection over 6 months	15568	1260	10149	11409
Diagnostically confirmed and treated malaria case per head population over 6 months	0.20	0.07	0.58	0.65
Diagnostic tests per head of population over 6 months	0.38	0.15	2.43	2.58
**Directly observed costs over 6 months**				
RDT tests conducted	29,376	2,652	42,556	45,208
Cost per test (US$)	0.31	0.31	0.31	0.31
**Total RDT cost (US$)**	**9,113**	**823**	**13,201**	**14,024**
Microscopy tests	521	n/a	n/a	n/a
Cost per test (US$)	1.30	n/a	n/a	n/a
**Total cost of microscopy (US$)**	**676**	**n/a**	**n/a**	**n/a**
AL treatments	21,827	1,279	10,684	11,963
Cost per treatment (US$)	1.38	1.38	1.38	1.38
**Total cost of AL (US$)**	**30,069**	**1,762**	**14,718**	**16,480**
Total personnel costs (US$)	281,150	18,001	18,001	18,001
Time commitment (% FTE)	30	10	90	100
**Personnel costs of malaria testing and treatment**	**84,345**	**1,800**	**16,201**	**18,001**
**Sundry maintenance, transport and running costs for six months (US$)**	**43,201**	**103**	**926**	**1,029**
**Total cost for Six months (US$)**	**167,404**	**4,488**	**45,046**	**49,533**
**Total non-treatment costs over 6 months (US$)**	**137,335**	**2,726**	**30,328**	**33,053**
**Projected annual summaries at observed rates of testing & treatment**				
**Total cost per head of population covered per year (US$)**	**4.31**	**0.51**	**5.14**	**5.65**
**Total non-treatment costs per head of population covered per year (US$)**	**3.53**	**0.31**	**3.46**	**3.77**
**Total cost per confirmed case treated (US$)**	**10.75**	**3.56**	**4.44**	**4.34**
**Projected potential summaries at optimized rates of active testing & treatment**				
**Total cost per head of population covered per year (US$)**			**10.68**	
**Total non-treatment costs per head of population covered per year (US$)**			**6.25**	
**Total cost per confirmed case treated (US$)**			**8.09**	

^vi^US$ 1 = ZMW 4.89, exchange rate has been rebased to fit current Zambian currency.

Source: http://www.boz.zm/(S(keg4bza3j0p4fx2uixtmnibq))/FinanciaMarkestReport.aspx. Accessed 1st October 2013.

^vii^FTE - Full Time Equivalent.

^viii^In the projected annual summaries, cost per head of population was calculated by dividing the total costs by the catchment population. The cost of non treatment is the cost of only testing such as in an elimination scenario. The cost per confirmed case if the total costs divided by the diagnostically confirmed and treated cases of malaria infection.

^ix^Projected potential summaries developed from assumptions that the HF and CHW passive will not change even in an optimized environment. Cost/head of population calculated by addition to observed cost of an average 9 missed active visits per person and 2 passive visits per person by the cost of RDTs and Treatment respectively. The cost per confirmed case treated is the total cost per head divided by the mean number of CHW detected infections per year of 1.3.

If community participation could be dramatically improved to ensuring the average resident is tested at least once per month, and the trend observed in Figure 4B is assumed to represent impact of frequent testing and treatment upon infection status, the cost and cost-effectiveness of detecting and treating this diminishing case load would approximately double, even in a pre-elimination scenario where improved vector control would negate treatment costs (Table 3).

## Discussion

Despite the fact that only a quarter of the covered resident population agreed to be tested in each monthly round of household visits by the CHWs, these active surveys by modestly remunerated paid CB staff identified >11,000 malaria parasite infections in a population of <18,000 residents in a single calendar year, of whom more than half were symptomatic in or around the time they were visited and may not have otherwise sought care. The far higher sensitivity with which these active household surveys by the CHWs detect cases of malaria infection is also reflected in the observation that this surveillance arm captured twice as high an incidence rate as the passive surveillance activities of the HFs and CHWs combined. The strong association of many symptoms, especially fever, headache and vomiting with malaria infection, particularly among individuals tested during active household visits by the CHWs, confirms previous reports that illustrate just how inaccurate the term *asymptomatic* is in relation to widespread chronic malaria infections [79-81] that clearly cause very large proportions of the overall burden of clinical illness in the community (Table 1, Figure 7). Clearly a large proportion of the population are infected with malaria, and suffering from a range of mild symptoms of clinical illness as a consequence, but do not feel ill enough to report to a HF or even to a nearby CHW to seek care. In addition to representing a major proportion of overall morbidity burden among the population, these chronic infections also act as a reservoir for continued transmission [4,5,27,80,82]. Regularly scheduled household visits by CHWs, who presumably will need to be paid for such a full time commitment, may therefore be extremely useful for identifying, treating and mapping the individuals who harbour chronic malaria infections and constitute the infectious reservoir that sustain transmission [38,83,84].

However, as implemented in this study, even these monthly active household visits, repeated on a continuous monthly survey cycle, had no obvious impact on malaria infection burden, possibly because most participants did not consent to testing often enough to benefit from any impact upon malaria infection and associated symptoms that are suggested, but not proven by, Figures 4 and 7, respectively. While the AL treatment used here has well-documented gametocidal properties [85-88], the limited sensitivity of RDTs or microscopy and considerable natural density fluctuations of circulating *P. falciparum* blood stages mean that approximately half of all malaria infections are sub-patent and escape detection by a single testing event [27,89,90]. Furthermore, mosquito-to-human transmission remained remarkably high in the study area, measured as a mean EIR of approximately 70 infectious bites per unprotected person per year [57,58]. The most likely explanation of the persistence of such intense transmission, despite reasonably high rates of LLIN use, supplemented with IRS in selected clusters towards the end of this study, is probably the emergence of pyrethroid resistance among local populations of *An. funestus*[91]. The high rates of re-infection suggested by these entomological surveys are consistent with and confirmed by the high rates of infection incidence [92] recorded here (Table 3) despite the imperfect sensitivity of RDTs [93,94]. Given the imperfect detection sensitivity of RDTs [93,94] and the rapid rates of re-infection that can be expected in a setting with such a high EIR [30,95-98], it is unsurprising that at least monthly screening and treatment is required to achieve dramatic reductions of malaria infection burden (Figure 4B), associated symptoms (Figure 7), and presumably transmission [27,99,100], even assuming these two figures reflect genuine impact rather than mere association. However, it is certainly encouraging that the apparent impacts among residents consenting to such frequent testing and treatment, which these associations suggest, compare very well with simulations and field data from annual mass screen and treat programmes [40], and even simulations and field observations of year-long mass drug administration programmes with treatment cycles of only four or even two weeks [19]. It may also be encouraging that, despite their known limited sensitivity, RDTs appear to be sensitive enough to detect persistent malaria infections if each individual is tested often enough (Figure 4B) so that the frequent sporadic surges of detectable parasitaemia characteristic of *P. falciparum* are captured [101,102]. If the observed association of parasitaemia with testing and treatment (Figure 4B) reflects genuine impact, this also suggests patient compliance with the AL treatment regime used in this study was probably comparable with high estimates (84.5%) from previous evaluations in Zambia [103].

Beyond extending delivery of diagnostic and therapeutic services to the grass roots community level, the CHWs also provided a remarkably informative source of surveillance data, including the overall burden and distribution of malaria and associated clinical symptoms, a number of important demographic and geographic determinants of risk, and the rates utilization of preventive interventions, such as IRS and ITNs. It is particularly encouraging that, despite their known sensitivity limitations, RDTs [93,94] appear to be more than adequate for monitoring disease burden through CHW extension systems that can guide programme implementation. Latent antigenaemia several weeks after successful clearance of infection can cause false positive results when using HRP2 based RDTs, and therefore over-prescription of anti-malarial drugs [104-106], so it is possible that estimates of cost and cost-effectiveness described in Table 3 may be improved upon with better diagnostic technology. If scale up of such CHWs is affordable beyond this research setting and could be scaled up across entire districts, provinces or even whole countries, such routinely collected data reported in disaggregated form from such small population subdivisions could be invaluable at all levels of programmatic monitoring and evaluation.

The costs of providing this CB extension of primary health care services, to provide both active and passive screening and treatment for malaria were substantive (Table 3), corresponding to 11.1% of the annual per capita health budget of Zambia in 2011 ($96) [107]. Furthermore, to achieve the full potential of this service by ensuring community-wide engagement in screening and treatment on at least a monthly basis (Figure 4B), these costs are likely to double, even if baseline levels of transmission were reduced to pre-elimination levels so that the costs of drug treatment were negated (Table 3). It is highly unlikely, or desirable, that such a cadre of CHWs would be mobilized to deal with surveillance and control of malaria alone so these CB personnel would also be required to deal with uncomplicated forms of other common illnesses like diarrhoea and pneumonia [51,54,108]. Even the passively provided malaria diagnosis and treatment services described here would need to be augmented with a range of other clinical services to be supported at programmatic level [51,54]. It is therefore difficult to envisage CHWs effectively or sustainably taking on such substantive commitments on a purely voluntary basis with no remuneration whatsoever. Barely more than a third of the overall costs of the active household surveys by the CHWs were accounted for by the cost of their meagre remuneration, and this figure would reduce to less than a fifth if the increased commodity costs of full compliance with monthly screening and treatment were to be incurred through improved community participation. Paying these CHWs is therefore not only likely to be essential to ensure their retention and effectiveness as full time agents of malaria infection surveillance and control, it is also a relatively minor fraction of the overall cost of actively delivering extended CB primary healthcare services.

Apart from its observational design, the most obvious limitation of this study is that the majority of enrolled residents were tested and, where appropriate, treated far less than once a month (Additional file 1, Figures 2A and C, 3C and D, and 4A) as originally envisaged. Future evaluations of CHWs, especially those engaged to conduct frequent active household surveys of their entire assigned populations, should include operational research studies to better understand and address the limitations of service uptake by community members observed in this study.

## Conclusions

The monthly active household visits to entire communities by CHWs equipped with existing field-compatible diagnostic tools were not sufficient to eliminate the human reservoir of malaria infection from this rural African setting with intense transmission despite reasonably high LLIN/IRS coverage. However, observed negative associations between infection status and frequency of testing and treatment suggest that dramatic impact upon malaria parasite infection risk and associated disease burden may be achievable through far more regular testing and treatment. Substantive alleviation of malaria may be attainable and cost-effective if the substantial, but not necessarily prohibitive, costs of implementing frequent active CB surveys for chronic malaria infections are affordable to national programmes and higher levels of community participation in regular testing opportunities are achievable.

## Abbreviations

AL: Artemether-lumefantrine; EIR: Entomological inoculation rate; CB: Community-based; CHW: Community Health Worker; HF: Health facility; IRS: Indoor residual house spraying; LLINs: Long-lasting insecticidal nets; RDT: Rapid diagnostic tests; GLMM: Generalized linear mixed model; FTE: Full time equivalent; NMCC: National Malaria Control Centre.

## Competing interests

The authors declare that they have no competing interests.

## Authors’ contributions

BH, AS, CHS, HM, MK and GFK: Conceived, designed and supervised all field activities of the study. AB provided the map of the study area. AB, TE and JM: Assisted in developing the data analysis plan. BH and GFK: Drafted the manuscript in consultation with the other authors, all of whom reviewed it and provided comments. All authors read and approved the final version of the manuscript.

## Supplementary Material

Additional file 1 Association of malaria infection with age, sex, symptoms, interventions, geographical location and season. Click here for file
