# Supplementary material for: Monitoring, characterization and control of chronic, symptomatic malaria infections in rural Zambia through monthly household visits by paid community health workers
Source: Malar J. 2014 Mar 31;13:128. doi: 10.1186/1475-2875-13-128 (PMC4113135; doi:10.1186/1475-2875-13-128)
Supplement: Additional file 1 — Association of malaria infection with age, sex, symptoms, interventions, geographical location and season. [file 1475-2875-13-128-S1.pdf]

Additional file 1. Association of malaria infection with age, sex, symptoms, interventions, geographical location and season [i,ii]

Category	Passive Surveillance				Active Surveillance			
	DP%[a]	n/N[b] (I)	OR [95%CI][c]	P[d]	DP%[a]	n/N[b] (I)	OR [95%CI][c]	P[d]
Overall	42.50	2236/5261(3804)	0.74[0.68,0.79]	<0.001	20.26	11851/58500 (17543)	0.25[0.23,0.27]	<0.001
Age								
<1	27.88	58/208 (145)	1.27[0.76,1.78]	0.352	12.82	204/1591 (635)	1.28[1.08,1.48]	0.015
1-4	42.11	480/1140 (768)	3.38[3.04,3.72]	<0.001	22.47	2211/9841 (2797)	3.03[2.92,3.15]	<0.001
5-10	59.10	799/1352 (935)	8.40[8.06,8.73]	<0.001	26.40	3794/14369 (3920)	4.16[4.04,4.27]	<0.001
11-14	53.55	294/549 (410)	7.05[6.68,7.43]	<0.001	25.19	1813/7198 (2265)	3.97[3.85,4.09]	<0.001
15-24	39.85	263/660 (510)	3.43[3.07,3.79]	<0.001	18.84	1690/8969 (3031)	2.26[2.14,2.38]	<0.001
25-44	27.32	244/893 (713)	1.81[1.46,2.17]	0.001	13.63	1470/10787 (3265)	1.36[1.25,1.48]	<0.001
≥45	20.41	79/387 (323)	1[NA][e]	NA[e]	11.70	617/5275 (1630)	1[NA][e]	NA[e]
Sex								
Male	44.32	1058/2387 (1699)	1[NA][e]	NA[e]	21.92	5802/26475 (7703)	1[NA][e]	NA[e]
Female	40.99	1164/2840 (2042)	0.83[0.67,0.99]	0.019	18.74	5901/31481 (8285)	0.86[0.81,0.92]	<0.001
Interventions								
ITN	39.02	1480/3793 (2738)	0.71[0.50,0.92]	0.002	17.14	7197/41978 (12295)	0.79[0.72,0.85]	<0.001
IRS	42.03	759/1806 (1224)	1.32[1.06,1.58]	0.036	15.39	2429/15788 (4323)	1.25[1.14,1.35]	<0.001
Number of preceding tests[f]	45.1[f]	2236/5261(3804)	0.92[0.90,0.93][f]	<0.001	30.7[f]	11851/58500 (17543)	0.91[0.88,0.95][f]	<0.001
Clusters								
Luangwa district								
Sinyawagora RHC	44.56	86/193 (178)	2.76[1.59,3.94]	0.090	23.23	809/3482 (1207)	1.75[0.96,2.54]	0.164
Kasinsa RHC	33.95	184/542 (454)	4.46[3.43,5.49]	0.005	13.51	684/5063 (1243)	2.39[1.61,3.18]	0.030
Chitope RHC	28.94	68/235 (179)	1.71[0.64,2.78]	0.325	17.58	1163/6614 (1030)	4.71[3.93,5.50]	<0.001
Luangwa High School RHC	44.14	644/1459 (732)	1.93[0.94,2.91]	0.191	18.57	1342/7225 (999)	2.08[1.30,2.86]	0.066
Mphuka RHC	39.10	217/555 (278)	2.79[1.72,3.87]	0.062	23.07	1249/5414 (1327)	1.59[0.81,2.37]	0.243

Mandombe RHC	36.22	205/566 (469)	1.22[0.22,2.22]	0.695	9.69	606/6252 (1206)	1.14[0.37,1.92]	0.734
Luangwa Boma RHC	22.03	39/177 (160)	1[NA][e]	NA[e]	5.90	368/6241 (1304)	1[NA][e]	NA[e]
Nyimba district								
Kacholola RHC	42.34	94/222 (194)	10.76[9.62,11.91]	<0.001	24.65	1198/4860 (1038)	6.87[6.09,7.66]	<0.001
Hofmeyer RHC	51.99	157/302 (280)	46.94[45.87,48.02]	<0.001	47.23	1050/2223 (1394)	13.25[12.46,14.04]	<0.001
Mtilizi RHC	66.94	164/245(193)	11.34[10.23,12.46]	<0.001	36.51	782/2142 (847)	5.93[5.16,6.70]	<0.001
Mtilizi RHP	50.00	19/38 (37)	9.24[7.89,10.58]	0.001	22.72	573/2522 (1227)	5.08[4.36,5.80]	<0.001
Chinambi RHC	57.35	39/68 (59)	10.44[9.01,11.86]	0.001	29.11	560/1924 (947)	6.90[6.02,7.78]	<0.001
Mkopeka RHC	54.90	112/204 (179)	12.03[10.89,13.16]	<0.001	35.41	1004/2835 (753)	6.27[5.43,7.11]	<0.001
Chipembe RHC	45.71	208/455 (333)	7.56[6.50,8.61]	<0.001	27.19	463/1703 (897)	5.89[5.07,6.71]	<0.001
Season								
Hot & wet (Dec – April)	50.47	1082/2144 (1865)	8.11[7.78,8.44]	<0.001	20.79	4615/22195 (9843)	4.92[4.71,5.13]	<0.001
Cool & dry (May - Aug)	46.26	929/2008 (1749)	4.99[4.67,5.31]	<0.001	25.38	6125/24137 (12025)	4.00[4.71,5.13]	<0.001
Hot & dry (Sept – Nov)	24.72	224/906 (858)	1[NA][e]	NA[e]	9.05	1091/12056 (7745)	1[NA][e]	NA[e]

[i] a – Diagnostic positivity, b – (n – Number RDT positive, N – Total number of testing events), I – number of individuals that participated , c – odds ratio with 95% confidence intervals, d – p-value ,NA [e] –Not applicable /reference group ,f – An integer continuous covariate so the diagnostic positivity presented represents that for the first visit calculated as the intercept of the model and the odds ratio presented represents proportional change of diagnostic positivity per additional preceding test.

[ii] The association of malaria infection with age, sex, symptoms, interventions, cluster, number of tests conducted per individual and season was determined using GLMM; with observed malaria RDT determined status as a binary dependent outcome with the independent categories of age, sex, symptoms, access and use of ITNs and/or IRS and seasons. The models included date and individual nested within CHW catchment nested within cluster as random effects except for one in which cluster was treated as a categorical variable to determine the effects of each cluster . Symptoms were removed from the final model for logical reasons, as they are effects and not causes of infection. The final model consisted of age, sex, access and use of ITNs and/or IRS, season, number of tests conducted per individual and geographical location as the determinants of malaria infection.
